# Rice husk ash and nanomaterial-blended cement composites: a review

**DOI:** 10.1007/s11356-025-37361-9

**Published:** 2026-01-12

**Authors:** Pasindu Samarajeewa, Samith Buddika, Hiran Yapa, Chamila Gunasekara, David W. Law

**Affiliations:** 1 Department of Civil Engineering, University of Peradeniya, Peradeniya, 20400 Sri Lanka; 2 Civil and Infrastructure Engineering, School of Engineering, RMIT University, VIC, 3000 Australia

**Keywords:** Rice husk ash, Nanomaterial, Cement composites, Sustainability, Durability, Construction materials

## Abstract

Enhancing the sustainability and performance of cement composites is crucial to the protection of the environment and advancing building technology. Numerous studies have been carried out to evaluate binary and ternary mixes incorporating micro- and nanosized supplementary cementitious materials (SCMs) to achieve these goals. Rice husk ash (RHA) stands out as an SCM due to its abundance as an agricultural residue having pozzolanic properties. However, the relatively low reactivity and the reduced workability due to the mesoporous structure hinder the adoption of RHA in cement composites. While nanomaterials offer the advantage of improved microstructure and higher reactivity, their high cost and demanding processing techniques limit their widespread adoption. Combined blends of nano- and micro-sized SCMs have shown promising results, as synergistic effects can mitigate their individual drawbacks, but the inter relationship is unclear. This paper reviews the existing studies on RHA and nanomaterial-blended cement composites to provide an understanding of their interactions. The available literature is categorized by the type of nanomaterial employed, and the mechanical, microstructural, and durability properties are summarized. The correlation between mechanical properties is derived, and summarized results are discussed to identify the underlying mechanism. Overall, this paper aims to provide a comprehensive overview of current knowledge on RHA-nanomaterial–blended cement composites. This summary will be beneficial in identifying research gaps and suggesting future directions for advancements in sustainable blended cement composites.

## Introduction

Concrete is utilized globally as a prime construction material due to its favorable engineering properties, low cost, and ease of use. As the traditional binding material of concrete, Portland Cement (PC) production and usage are escalating rapidly in line with global infrastructure development. Annual cement consumption in 2023 was around 4.1 billion tons, and it is predicted to reach 4.7 billion tons by 2050 (Schneider 2019). Production of cement has a very high environmental impact, responsible for about 8% of global CO_2_ emissions. Because of this, researchers have been exploring potential supplementary materials as alternative binders to PC. The earliest supplementary cementitious materials (SCMs) to be adopted include fly ash, pozzolana, micro silica, and ground granulated blast-furnace slag (Meraz et al. 2023). More recently, other alternatives that can replace PC have also been identified, e.g., geo-polymer binders, alkali-activated slag. Furthermore, the potential of utilizing agricultural by-products to replace partially PC has also been recognized.

In the use of agricultural by-products in the construction industry, the options identified include rice husk ash (RHA), sugarcane bagasse ash (SCBA), and palm oil fuel ash (POFA). RHA, which is a byproduct of rice production, is abundantly available in rice-producing countries. Rice husks are used as a biofuel for electricity generation, and RHA applications include low-level fertilizer, cosmetic ingredients, and absorbents. However, the majority is dumped as waste or in landfill. Global rice production reached 540 million metric tons in 2024 (FAO^2024^), which can potentially generate 35 million tons of RHA annually. RHA, after combustion, possesses a high silica (SiO_2_) content ranging from 80 to 99% in the amorphous phase (Fernando et al. 2023; Ganesan et al. 2008; Huang et al. 2017; Nduka et al. 2022).

When RHA is incorporated into cement, the silica in RHA reacts with calcium hydroxide (CH) to form calcium silica hydrate (CSH) gel (Jayaraman et al. 2023). CSH is the cornerstone material responsible for the mechanical strength and durability of cement composites. RHA incorporation in cement composites has been shown to improve both mechanical and durability properties. However, the replacement level for RHA is typically limited to 10–20% to maintain optimal fresh and hardened properties (Abalaka 2013; Ganesan et al. 2008; Jayaraman et al. 2023). Investigating methods to achieve greater RHA replacement ratios while maintaining these properties could significantly reduce cement content, promoting sustainability in the construction industry. It is however of note that the physical and chemical properties of RHA can vary depending on burning conditions, grinding procedures, climate, soil chemistry, and the type of fertilizer used. Chemical composition, phase, and organic impurity content also significantly influence the properties of cement composites (Kwan and Wong 2020; Siddika et al. 2021).

The construction material field is witnessing a growing interest in nanomaterials. The inclusion of nanomaterials has been shown to improve the attributes of concrete in several ways, including filling pores within the matrix, refining the microstructure, and reacting with other components to produce beneficial hydration products that contribute to overall performance. A small addition of these nanomaterials can significantly enhance the properties of the composite. However, the influence of nanomaterials on the properties of cement composites depends on the chemical and physical characteristics of the particles. Nanomaterials can be categorized based on their dimensionality: nanoparticles (zero-dimensional), fibers (one-dimensional), and platelets (two-dimensional, 2D) (Chuah et al. 2014). The shape and morphology significantly impact the characteristics of these particles. Graphene oxide (GO), reduced graphene oxide (rGO), and graphene nanoplatelets (GNPs) are 2D sheet-like nanocarbon materials, while nanosilica (NS), nano-TiO_2_ (NTiO_2_), and nano-CaCO_3_ (NCaCO_3_) exist as particles. Despite the promising potential of nanomaterials in cement, several challenges remain. One major concern is their impact on workability. The high surface area of nanoparticles can lead to the absorption of free water in the mix, hindering workability. Additionally, van der Waals forces between nanoparticles can cause them to agglomerate, further hindering their uniform dispersion within the cement matrix. However, the incorporation of these nanomaterials with other SCMs to form ternary and quaternary mixes may prove to be a promising approach to overcoming limitations and potential drawbacks associated with individual SCMs.

While extensive research on RHA-blended cement composites has been conducted (Amin et al. 2022; Endale et al. 2023), a significant number of studies are now emerging with reference to RHA-nanomaterial blended composites. These nanomaterials encompass nanosilica, nanocarbon, nano-RHA (NRHA), NTiO_2_, NCaCO_3_, and nano-CuO (NCuO) (Anto et al. 2022; Chuah et al. 2014; Shaikh and Supit 2014). It has been observed that each nanomaterial generally interacts uniquely with the composite. Researchers have primarily evaluated these composites based on their mechanical properties, durability, and microstructure but have not addressed their inter-relationship in detail.

In this context, this paper reviews the critical outcomes of all such research. Comparisons across results are made; overall benefits and barriers of RHA-nanomaterial applications are identified. The compilation of this information will be useful in promoting the application of ternary composites in the future construction industry.

In order to preserve consistency, a composite where cement is replaced with X% of RHA or Y% of nanosilica (NS) will be denoted as RHA-X or NS-Y respectively. A ternary mix of both materials will be represented as RHA-X + NS-Y (NS: nanosilica, NC: nanocarbon, NCuO: nano-CuO, NTiO_2_: nano-TiO_2_, NRHA: nano-RHA, and NcaCO_3_: nano-CaCO_3_).

## Methodology

Numerous studies have investigated the use of RHA as a SCM in cement composites, demonstrating its potential to enhance various properties of the concrete produced. Similarly, studies on the inclusion of nanomaterials in concrete have demonstrated their potential to enhance performance. Hence, the incorporation of nanomaterials alongside RHA in ternary blends presents a promising avenue for further improving the performance of cementitious composites. While existing research has shown potential benefits, the optimal replacement levels and application conditions for these combined mixes remain elusive.

This research paper aims to bridge this gap by establishing a comprehensive database of the properties of nanomaterials and their impact on the performance of nanoblended cementitious composites containing RHA. This knowledge will facilitate further exploration and development of these advanced materials.

To achieve this objective, a systematic literature review was conducted using the Scopus database. The search criteria included keywords related to “rice husk ash,” “cement composites,” and various “nanomaterials” (e.g., nanocarbon, nanosilica, and nano-TiO_2_). The search was limited to peer-reviewed journal articles published between 2013 and 2024.

**Fig. 1:**
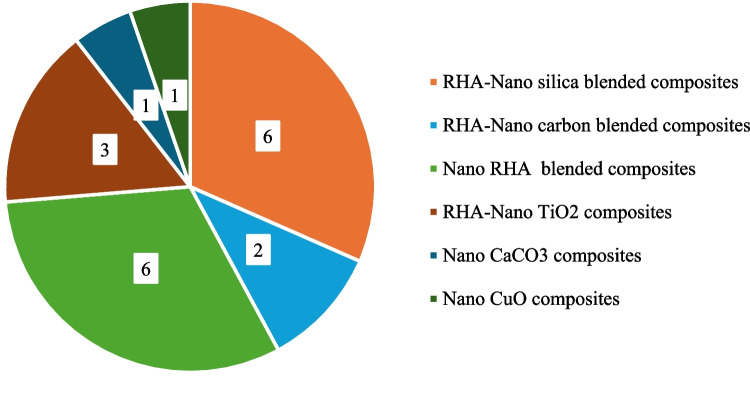
Published studies on RHA and nanomaterial-blended cement

**Fig. 2:**
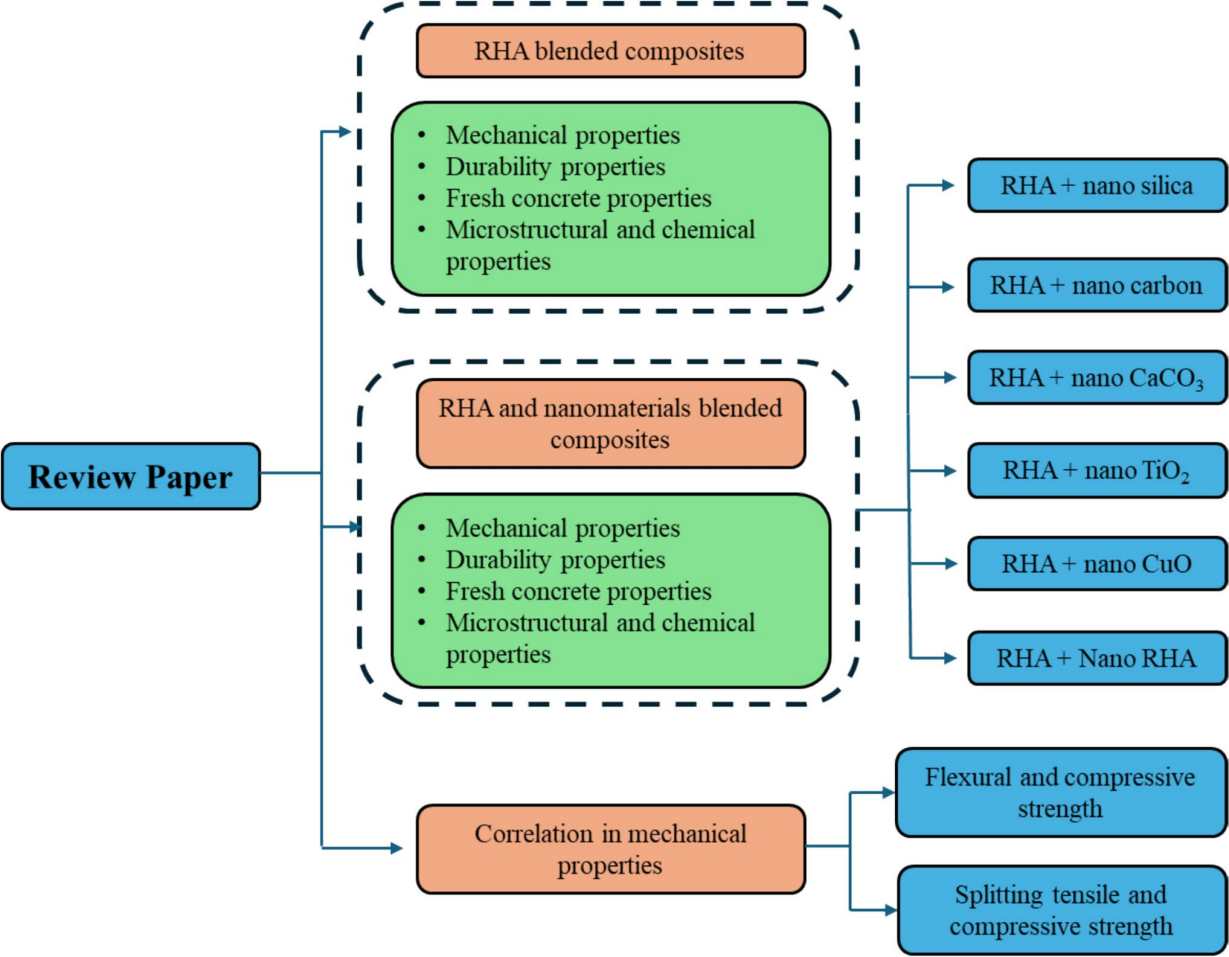
Key components of the review paper

The initial search results were meticulously screened to remove irrelevant papers. This manual filtering process resulted in the selection of 19 relevant research articles for in-depth analysis. Figure 1 illustrates the number of studies of RHA and nanomaterial-blended composites included in the review. Figure 2 depicts the key components discussed in the review paper.

**Fig. 3:**
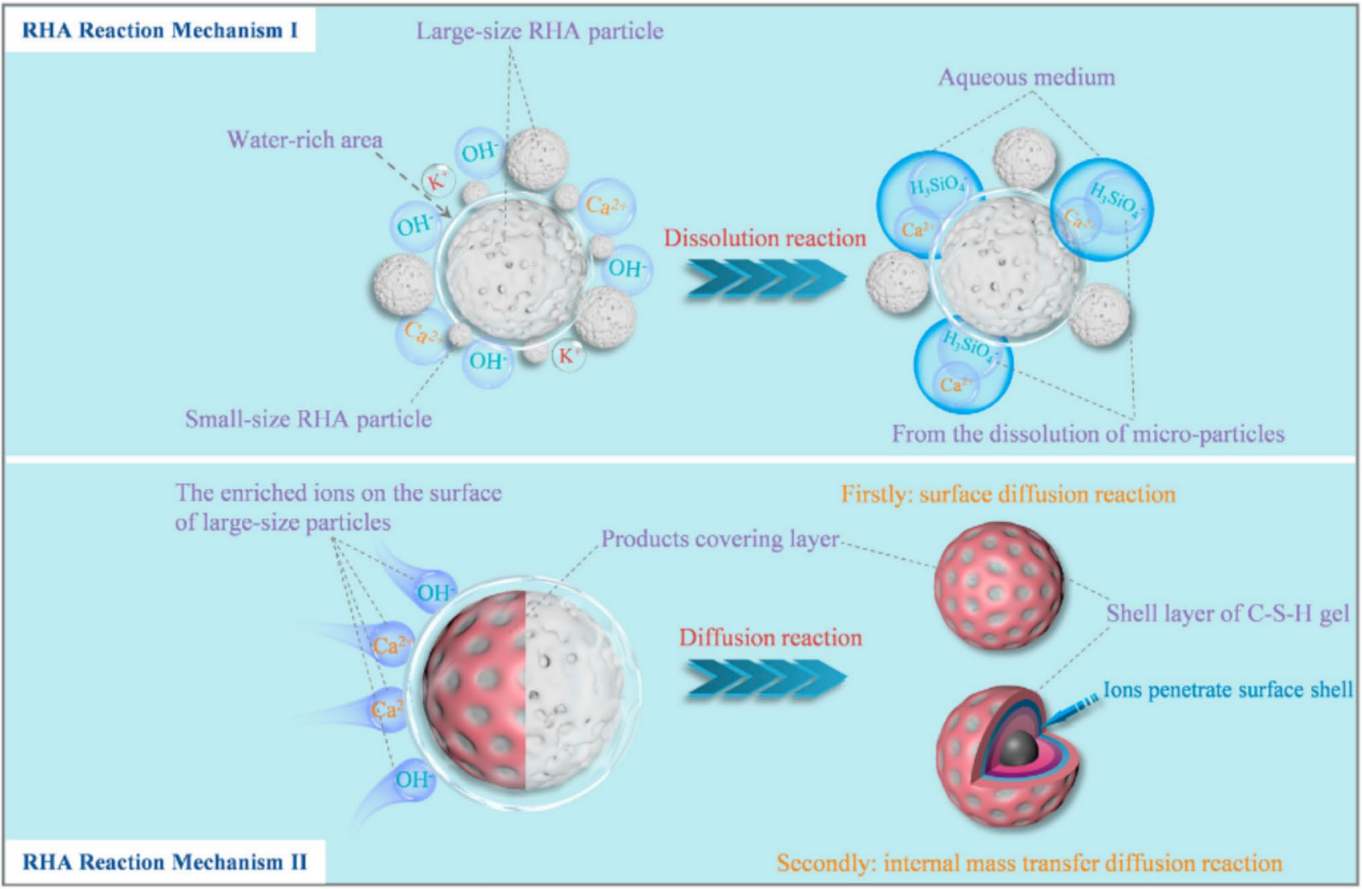
Reaction mechanism of RHA in cement (Wang et al. 2024)

**Fig. 4:**
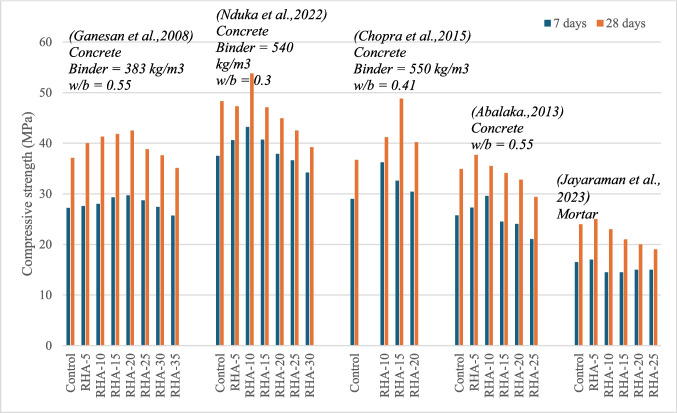
Variation of compressive strength of RHA-blended cement composites

## RHA-blended composites

RHA acts as a natural pozzolanic material in cement composites. In the cement matrix, the SiO_2_ in RHA primarily reacts with Ca(OH)_2_ to form the cementitious product, CSH gel. Initially, the SiO_2_ in RHA dissolves under alkaline conditions by reacting with OH^−^ ions to form silicic acid ions (H_3_Si${\mathrm{O}}_{4}^{-}$). Subsequently, Ca^2+^ ions react with these silicic acid ions to form the C-S-H gel product (Wang et al. 2024). The reaction mechanism is illustrated in Fig. 3.

The mechanical properties of RHA-blended cement composites exhibit some variability, likely due to the differing characteristics of RHA (Abalaka 2013; Jayaraman et al. 2023). Figure 4 summarizes the compressive strength results of studies on RHA-blended cement composites. It is observed that optimal compressive strength is achieved at RHA replacement levels of between 10 and 20%. Compressive strengths resulting from evaluated studies show a 4–32% increase at 28 days with the inclusion of RHA (Abalaka 2013; Chopra et al. 2015; Ganesan et al. 2008; Jayaraman et al. 2023; Nduka et al. 2022). Similar trends are observed in splitting tensile strength and flexural strength tests (Ganesan et al. 2008; Kwan and Wong 2020).

RHA addition to cement mixes typically reduces flowability as the RHA percentage increases. This limits the quantity of RHA that can be effectively used or requires the addition of superplasticizers (SP) or extra water to maintain workability. These phenomena are attributed to the porous, irregular, and angular shape of RHA particles. Their porous nature allows them to absorb water, reducing the free water available for initial hydration (Siddika et al. 2021).

It is observed that water absorption of concrete is reduced with RHA replacement (Sandhu and Siddique 2017; Zareei et al. 2017). Nevertheless, absorption varies with the water/binder ratio, and some studies report an increase in water absorption with the level of RHA replacement. This may be due to the characteristics of the specific RHA (Mehdizadeh et al. 2021).

The inclusion of RHA has been observed to affect the durability properties. Salas et al. (2009) observed a 60% reduction of the Rapid Chloride Penetration Test (RCPT) value for the RHA-10 mix, while Zareei et al. (2017) found a 78% reduction of the RCPT value for the RHA-25 mix compared to the control mixes. Hu et al. (2020) evaluated the reduction in compressive strength of samples subjected to sulphate attack and observed a 17.7% improvement for the RHA-15 mix compared to the control mix at 120 days. This enhancement of durability properties is attributed to the reduced porosity and pore connectivity of the composite (Mamun and Islam 2017). Kannan and Ganesan (2014) observed that RHA-blended concrete exhibits a more uniform structure compared to a conventional mix, as shown by scanning electron microscopy (SEM). Increased formation of CSH gel due to the pozzolanic reaction of RHA refined the pore structure. However, variations in performance are observed across different studies, making it difficult to correlate performance with replacement level. These variations are primarily attributed to the mix proportions and characteristics of RHA. The characteristics of RHA depend on several factors, including burning conditions, grinding procedures, climate, soil chemistry, and the type of fertilizer used (Kwan and Wong 2020; Siddika et al. 2021).

Among these factors, burning and grinding procedures have been noted to significantly influence the chemical composition, phase, and organic impurity content, thereby affecting the performance of blended cement composites. Rice husks burned at temperatures between 500 and 700 °C exhibit higher silica content in the amorphous phase, which readily participates in the pozzolanic reaction (Nair et al. 2008; Zain et al. 2011).

## RHA-nanomaterial composites

There are limited studies on RHA-nanomaterial blended composites. Among these, RHA -nanosilica-blended composites have garnered significant research attention. Several studies have focused on composites incorporating nanocarbon materials such as GO and GNP, as well as other materials including NCaCO_3_, NTiO_2_, and NCuO blended with RHA. Additionally, some studies have evaluated the performance of nano-RHA-blended composites in binary and ternary mixtures. These studies primarily investigate the fresh, mechanical, and durability performance of composites while examining variations related to the characteristics of these SCMs.

The reaction mechanisms of nanomaterials in cement composites are primarily dictated by their chemical composition and their reactivity with the existing cementitious phases. For instance, chemicals such as SiO_2_, TiO_2_, and CuO engage in a pozzolanic reaction, which is crucial for producing additional CSH, a key strength-contributing component (Anto et al. 2022; Chuah et al. 2014). In contrast, CaCO_3_ undergoes a direct hydration reaction, while the carboxylic acid groups present in nanocarbon materials function as accelerators, increasing the overall hydration process of the cement.

Furthermore, both the size and morphology of the nanomaterials significantly influence the ultimate performance of the cement composite (Chuah et al. 2014). Specifically, the 2D structure of nanoplatelets (GO) is particularly effective in enhancing the tensile and flexural strength. Conversely, spherical nanoparticles tend to improve the microstructure primarily through a dense-packing effect and by exhibiting a higher intrinsic reaction rate compared to micro-sized materials.

### RHA-nanosilica (RHA-NS) composites

In contrast to micro silica, NS-blended applications exhibit lower optimal replacement percentages, i.e., up to 3% (Anto et al. 2022). This is primarily due to the combined effects of greater reactivity and denser packing of NS particles. The use of higher NS dosages often leads to agglomeration, which is detrimental to performance. Hence, it is crucial to consider the factors that influence performance, such as particle size, surface area, the specific type of nanomaterial, and the dosage employed.

#### Mechanical properties

**Fig. 5:**
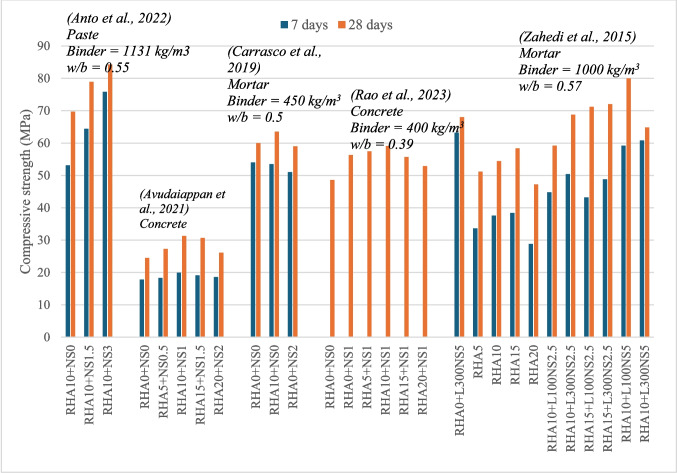
Variation of compressive strength of RHA and nanosilica-blended cement composite (L300NS and L100NS denote NS with SSA of 100 m^2^/g and 300 m^2^/g accordingly)

Figure 5 summarizes the compressive strength results of RHA + NS-blended composites. Anto et al. (2022) investigated the effect of RHA-10 with 0–3% NS in concrete at 0.35 and 0.55 water-to-binder (w/b) ratios. The RHA-10 + NS-3 mix resulted in 14.2% and 20.9% compressive strength increments, respectively, for 0.35 and 0.55 w/b ratios. Avudaiappan et al. (2021) incorporated NS between 0 and 2% in 0.5% intervals, while progressively increasing RHA from 0 to 20% in 5% intervals. The RHA-10 + NS-1 mix achieved the optimal compressive strength, with a 27.7% increase, while all mixes exhibited enhanced compressive strength compared to the control. NS-blended mixes also demonstrated higher early-age strength.

According to Andrade et al. (2020), concrete containing RHA-10 + NS-10 achieves a 29% increase in compressive strength, compared to only a 3% increase for RHA-10 alone. Zahedi et al. (2015) observed that NS with higher SSA exhibited higher early-age strength, while lower SSA resulted in higher long-term strength at 90 days. Overall, ternary mixes yielded better results than binary mixes. Notably, RHA-10 + NS-5, possessing lower SSA, demonstrated the optimal performance for both 28 and 90 days. 

Rao et al. (2023) evaluated the performance of ternary concrete mixtures containing 0–20% RHA with 1% NS as well as binary mixtures containing 1–5% NS. Their results indicate that the RHA-10 + NS-1 mix achieved a 22% increase in compressive strength, while the binary mix with NS-3 exhibited a 43% increase. However, this study did not explore higher NS percentages in combination with RHA and did not establish an upper limit for NS addition.

Anto et al. (2022) observed that the flexural strength of the mortar mix followed a similar trend to the compressive strength**.** This finding is further supported by Avudaiappan et al. (2021) and Rao et al. (2023), who also studied the correlation of compressive and flexural strength, as discussed in depth in the section on the correlation in mechanical properties. Similarly, the splitting tensile strength of mixes containing RHA and NS exhibited a trend consistent with the other mechanical properties observed.

#### Fresh concrete properties

Avudaiappan et al. (2021) noted that the blending of RHA and NS into concrete reduces workability. This decrease is primarily attributed to the high surface area of NS particles, which leads to water absorption and a reduction in the water available for initial hydration. To maintain workability in cement pastes containing RHA and NS, Andrade et al. (2020) employed varying dosages of SP. Their results showed that 1.05% and 0.14% SP were required for the NS-3 and RHA-10 mix, respectively. Additionally, the combined RHA + NS mix necessitated an even greater SP dosage.

Zahedi et al. (2015) investigated the influence of particle size and SSA of NS on workability and found that NS with lower particle size and higher SSA required a larger quantity of SP to maintain consistent workability. Further research is required to clarify the individual effect of materials when combined in a ternary mix.

Liu et al. (2025) examined the hydration kinetics of RHA and NS-blended cement paste. They observed that NS reacts at lower temperatures (around 20 °C), while RHA reacts at higher temperatures. Additionally, the higher reactivity of NS tends to release more heat at an early age, whereas the RHA-blended mix releases heat at later stages. Overall heat generation is also lower for the blended mixes because the pozzolanic reaction produces less heat.

#### Durability properties

Anto et al. (2022) reported that incorporating NS decreased the water absorption of pastes while increasing the bulk density. This indicates a reduction in voids compared to mixtures containing only RHA. Ultrasonic Pulse Velocity (UPV) test results by Anto et al. (2022) demonstrate that higher NS content correlates with higher pulse velocities, indicating improved quality and performance.

Carrasco et al. (2019) observed a reduction in porosity in RHA and NS-blended mortar mixes compared to the control mix, with the RHA-blended mix exhibiting the lowest porosity at 7 and 28 days. Micro- and nanosized SCMs may be responsible for this reduction. The study also suggested that RHA may lead to lower porosity due to increased reactivity resulting from the even distribution of particles, while agglomeration of nanosilica reduces the hydration reaction, making porosity higher compared to the RHA-blended mix. Furthermore, this RHA-blended mix contained capillary pores with small diameters, which reduced pore connectivity and consequently hindered the penetration of water or other harmful substances.

Zahedi et al. (2015) investigated chloride migration and observed that RHA-blended mixes had reduced permeability. However, ternary mixes containing nanosilica exhibited an even greater reduction, potentially due to a refined pore structure. Furthermore, the study suggests that fine nanosilica particles contribute to early age development, while coarser particles exhibit improvements at later ages (between 28 and 90 days). Notably, the RHA-10 + NS-5 mix showed a significant improvement compared to the control mix.

Anto et al. (2022) investigated the effect of acid attack by comparing the mass loss of cement composites exposed to acid and found that the RHA-10 + NS-3 mix significantly reduced mass loss, equivalent to 27.5% compared to the RHA-10 mix.

#### Microstructural and chemical properties

Avudaiappan et al. (2021) employed SEM images and image processing techniques to analyze the pore characteristics of concrete blended with RHA and NS. The study evaluated the optimal mix (RHA-1 + NS-1) with and without sonication, together with a control mix. Their observations revealed that the mix containing RHA and sonicated NS exhibited the lowest porosity and pore radius, while the mix with only RHA displayed the highest. This finding suggests that the performance of cement composites can be enhanced by employing proper mixing methods for nanomaterials.

Rao et al. (2023) investigated the microstructure of concrete containing 10% and 20% RHA with 1% NS using SEM. They observed that higher RHA replacement resulted in a denser structure with fewer pores. Additionally, NS appeared to contribute to the formation of additional CSH and a more refined pore structure. Furthermore, X-ray diffraction (XRD) analysis at 28 days revealed the presence of tobermorite in RHA-blended mixtures, indicating an ongoing pozzolanic reaction.

Givi et al. (2013) found that the pore structure becomes increasingly refined as the percentage of NS increases in the mix. Andrade et al. (2020) observed that ternary mixes containing both RHA and NS exhibited an even greater degree of pore refinement compared to binary mixes containing only RHA or NS.

Thermogravimetric analysis (TGA) was employed by Andrade et al. (2020) to quantify the CH content in the mixes. Their findings revealed a significant reduction in CH for the RHA-10 + NS-3, with a decrease of 36% and 55% at 28 and 90 days, respectively. This reduction can be attributed to the pozzolanic reaction. In contrast, all binary mixes exhibited a minimal reduction in CH content (less than 25%). Fourier-transform infrared spectroscopy (FTIR) results corroborated the TGA findings, indicating lower CH content and higher CSH content in ternary mixes. Furthermore, they observed the pozzolanic reaction was proportional to the amorphous silica content.

### RHA-nanocarbon (RHA-NC) composites

**Fig. 6:**
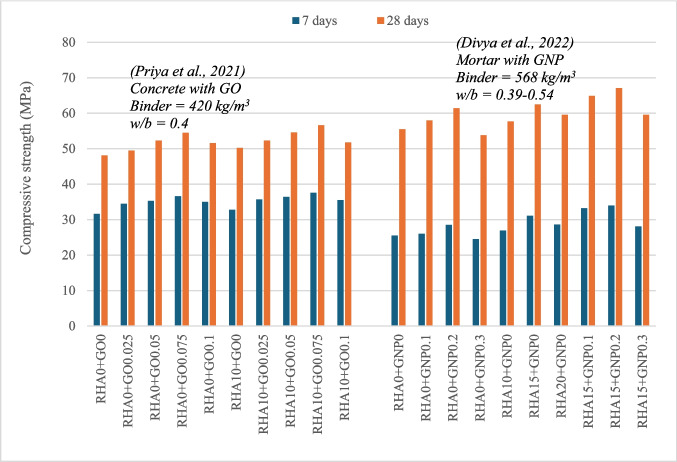
Variation of compressive strength of RHA and nanocarbon-blended cement composites

The performance of nanocarbon-blended composites is heavily influenced by the specific type of nanomaterial used. Figure 6 summarizes the compressive strength of RHA and nanocarbon-blended composites in previous studies. Priya et al. (2021) examined the properties of concrete combined with RHA and GO, incorporating GO up to 0.1% with 10% RHA. Compared to the control mix, the binary concrete mix with GO-0.075 displayed a 13% improvement in compressive strength, while RHA-10 + GO-0.075 achieved an increase of 17.6%. For flexural strength, those mixes with the highest compressive strength show a 26.8% and 29.7% increase, while for splitting tensile strength it is 21.2% and 34.3%. GO possesses a higher SSA and exhibits a strong affinity for water. This translates to an increased water requirement to maintain the desired workability of the mix. Fonseka et al. (2023) observed an 81% reduction in slump when incorporating 0.08% GO, highlighting this demand for water.

All mixes containing RHA and GO exhibited lower water absorption and sorptivity compared to the control mix (Priya et al. 2021). The RHA-10 and GO-0.075 binary mix showed the most significant reductions, with a 14.5% and 25.4% decrease in absorption, respectively. Interestingly, the RHA-10 + GO-0.075 ternary mix displayed an even greater reduction of 41.7% in absorption. However, further increasing the GO content led to a rise in absorption, potentially due to particle agglomeration and uneven dispersion.

It is observed that all mixes containing GO and RHA exhibited greater resistance to chloride penetration compared to the control mix without these additives. Similar to the trends observed for other mechanical and durability properties in this study, the RHA-10 + GO-0.075 mix displayed the greatest resistance at both 28 and 90 days. This enhanced resistance can be attributed to the interconnected layers of graphene, which act as a barrier, hindering the penetration of chloride ions. Additionally, the pozzolanic activity of both RHA and GO contributes to further improvement in resistance, with results at 90 days demonstrating continued improvement. However, Demircilioglu and Teomete (2024) found that the electrical resistivity of concrete with graphene and carbon nanotubes increases due to conductive phases in the materials. No studies have been reported on the electrical resistivity of RHA and nanocarbon materials.

Priya et al. (2021) investigated the microstructure of concrete containing RHA and GO. Their observations revealed that GO enhanced the properties of the mix by promoting a denser structure. The study further proposes that CSH gel and the carboxylic acid groups present in GO can react, potentially accelerating the hydration process. Compared to mixes containing only RHA, the ternary mixes exhibited a more intricate microstructure, suggesting a reinforcement effect provided by the GO. Energy-dispersive X-ray spectroscopy (EDAX) analysis confirmed a higher silica content in the ternary mix, likely due to the formation of a larger quantity of CSH gel.

Divya et al. (2022) investigated the combined effect of RHA and graphene nanoplatelets (GNPs). They varied the GNP content from 0.1% to 0.3% in mortar mixes containing 15% RHA. The RHA-15 + GNP-0.2 mix achieved the highest compressive strength, exceeding the control mix by 20.9%. This improvement was significantly greater than that observed when RHA and GNPs were added separately. It was also found that less additional water was needed to achieve consistent workability for mortar blended with RHA and GNPs. This discrepancy can be attributed to the use of SPs during graphene synthesis.

Divya et al. (2022) employed SEM and XRD analysis to investigate composites containing GNP and RHA. Their findings suggest that the negatively charged graphene particles attract SiO_2_ and Calcium Oxide (CaO) present in the RHA-blended cement, promoting the dispersion of graphene throughout the mix. This phenomenon leads to a denser cement matrix and improves physical characteristics. Compared to the control mix, the GNP mix exhibited a reduction in calcite (CaCO_3_) due to reduced carbonation and accelerated hydration of the cement paste. Furthermore, the addition of RHA resulted in a decline in portlandite peaks, indicating a higher tobermorite content, which is an initial phase of CSH gel.

### RHA-nano-CaCO_3_ composites

**Fig. 7:**
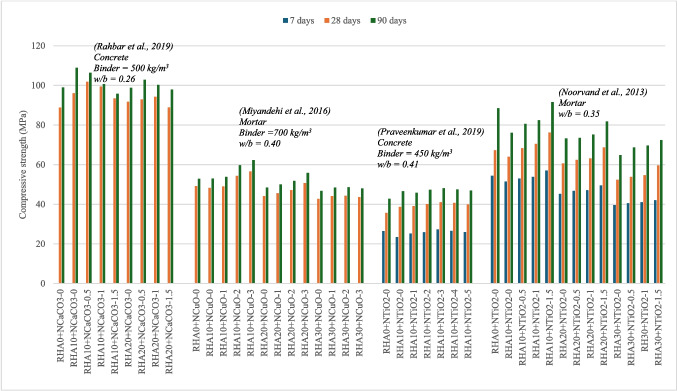
Variation of compressive strength of RHA and nano-CaCO_3_-, nano-CuO-, and nano-TiO_2_-blended cement composites

Rahbar et al. (2019) investigated the effect of incorporating NCaCO_3_ 0–1.5% with 10–20% RHA in concrete. The RHA-10 + NCaCO_3_−0.5 and RHA-20 + NCaCO_3_−1 mixes achieved the highest compressive strength, with 15% and 6% improvement, compared to the control mix, as shown in the Fig. 7. According to Shaikh and Supit (2014), NCaCO_3_ particles can react with tricalcium aluminate (C_3_A) and tricalcium silicate (C_3_S) in the cement, leading to increased hydration products, accelerated setting, and enhanced early strength development. However, the study does not explicitly attribute the observed improvements solely to these variations in nanoparticles.

It was also noted that combining RHA and NCaCO_3_ resulted in decreased workability, and a higher SP dosage was required to maintain consistent workability. Incorporating NCaCO_3_ with RHA reduced water absorption by around 10–20% across all test specimens. Furthermore, the study observed a 20–65% increase in electrical resistivity for both binary and ternary blended mixes compared to the control mix. However, the positive effect on these durability properties became less pronounced as NCaCO_3_ content increased from 0.5 to 1.5%.

This study also evaluated the mass loss and strength reduction of the mixes after exposure to acid attack for 28 and 84 days. Compared to the control mix, the RHA and NCaCO_3_-blended mixes exhibited lower mass loss and strength reduction at 28 days. Interestingly, these differences were not observed at the longer immersion time of 84 days.

Rahbar et al. (2019) further employed the silver nitrate (AgNO_3_) spray method to assess chloride ion penetration. Their results indicated that all RHA and NCaCO_3_-blended mixes displayed reduced chloride ion penetration compared to the control mix. Notably, incorporating NCaCO_3_ further reduced the penetration depth, likely due to a decrease in porosity and reduced conductivity. The study observed approximately 20% higher resistance to chloride penetration in the ternary mix with NCaCO_3_−1.5 compared to the corresponding binary mix containing only RHA.

Gopalakrishnan et al. (2025) evaluated the compressive strength of cement mortar with 10–40% RHA and 1–3% NCaCO_3_. All mixes exhibited improvement, while RHA-30 + NCaCO_3_−3showed the highest compressive strength, with increases of 69.3%, 40.8%, and 39.2% observed at 7, 28, and 90 days, respectively.

Water absorption was reduced up to the 30% RHA mix, with RHA-30 + NCaCO_3_−3 having the lowest absorption. Electrical resistivity and chloride permeability (using the RCPT) followed a similar pattern, and RHA-30 + NCaCO_3_−3 had a 57% reduction in the RCPT value (Gopalakrishnan et al. 2025).

This behavior is likely due to the pozzolanic reaction and the pozzolanic effect of nanoparticles. SEM images highlighted that the addition of RHA-30 + NCaCO_3_−3 densified the microstructure and reduced the large capillary pores, which contribute to the enhanced mechanical and durability performance.

#### RHA-nano-CuO composites

Miyandehi et al. (2016) investigated the addition of 1–3% CuO nanoparticles (NCuO) in combination with 10–30% RHA in cement mortar. The RHA-10 + NCuO-3 mix achieved the highest compressive strength at 28 days, exhibiting a 15.2% improvement compared to the control mix, Fig. 7. Interestingly, RHA-20 + NCuO did not show a significant increase, while RHA-30 + NCuO mixes displayed a decrease in strength of approximately 10% for all NCuO additions when compared to the control mix. Additionally, the strength at 90 days did not show a significant change compared to the 28-day results. UPV tests gave higher velocities for all mixes compared to the control mix, attributed to the improved packing density of the composite material due to the presence of nanoparticles (Miyandehi et al. 2016).

NCuO did not appear to significantly affect workability, as the same amount of SP was used for all NCuO content, while the SP percentage was increased for RHA. Mortar mixes containing RHA and NCuO demonstrated enhanced electrical resistivity for both ternary and binary mixes compared to the control mix. However, the addition of RHA resulted in a substantial increase in resistivity, with NCuO further enhancing this value. Notably, incorporating more than 20% RHA led to a decrease in resistivity, suggesting the presence of excess pozzolanic material that is not effectively contributing to the cement matrix. Similar trends were observed for chloride permeability, which correlated with electrical resistivity. The RHA-10 + NCuO-3 and RHA-20 + NCuO-3 mixes displayed increases in resistance of 35% and 52%, respectively.

Water absorption tended to increase for mixes containing more than 10% RHA, exceeding the value of the control mix for the 30% RHA mix. However, the incorporation of NCuO significantly reduced water absorption. This behavior of RHA can be attributed to its irregular shape and porous structure, which can trap water and impede the hydration process, resulting in the formation of permeable voids.

### RHA-nano-TiO_2_ composites

Praveenkumar et al. (2022, 2019) evaluated the effects of concrete incorporating 1–5% NTiO_2_ with 10% RHA. There was no improvement in strength at 7 days; however, strength did increase at 28 and 90 days. The RHA-10 + NTiO_2_−3 mix achieved the optimal performance, exhibiting a 15% improvement in compressive strength at 28 days. Similar trends were observed for splitting tensile and flexural strength.

The RHA and NTiO_2_-blended concrete mixes had lower chloride permeability compared to the control sample. At 28 days RHA-10 had a 5% reduction, which increased to 35% with the addition of NTiO_2_. Furthermore, blended concrete specimens exhibited less weight loss under acid attack compared to the control mix, with the ternary mix demonstrating the optimum performance.

Hassan et al. (2013) investigated the performance of cement mortar containing 10–30% of black RHA (BRHA) with the addition of up to 1.5% NTiO_2_. Their findings showed that RHA-10 + NTiO_2_−1.5 and RHA-20 + NTiO_2_−1.5 mixes displayed improvements of 13% and 2% in strength. This suggests that NTiO_2_ can potentially help maintain the performance of the composite by reducing the detrimental effects of untreated BRHA. However, RHA-30 + NTiO_2_−1.5 resulted in an 11% reduction in strength.

It was noted that the RHA-10 mix exhibited reduced flowability with the addition of NTiO_2_, while RHA30 mixes showed improved flowability with increasing NTiO_2_ content. This phenomenon can be explained by the potential of NTiO_2_ particles to fill the surface pores of the RHA particles, thereby reducing the amount of water absorbed by the RHA itself. These studies suggest that NTiO_2_ particles can act in two ways: as a pozzolanic material contributing to additional hydration products and by densifying the microstructure by filling nano- and micro-pores within the concrete.

### Nano-RHA composites

Nano-RHA can be obtained from RHA via either chemical synthesis (Ashok et al. 2017; Lim et al. 2018; Praveenkumar and Vijayalakshmi 2019) or grinding (Balapour et al. 2017; Mostafa et al. 2020; Tran et al. 2021). It was observed that chemical synthesis results in NRHA with a higher silica content, while grinding only reduces particle size.

**Fig. 8:**
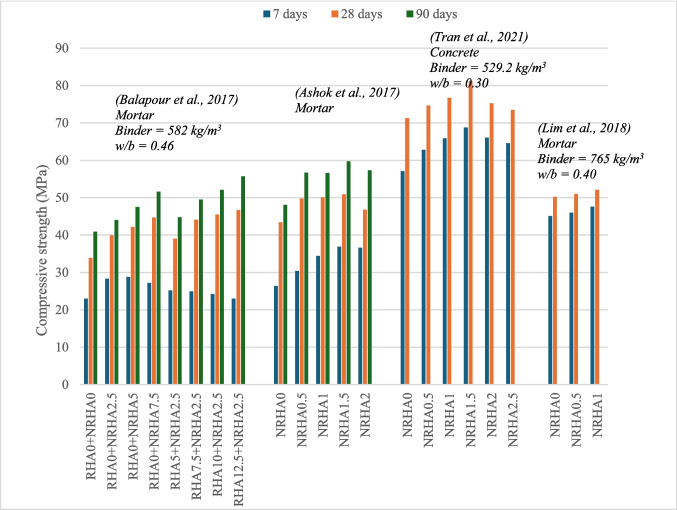
Variation of compressive strength of RHA and nano-RHA-blended cement composites

Mostafa et al. (2020) compared the properties of concrete containing NRHA and micro-RHA (MRHA) over a range of replacement levels (0–10%). Their findings indicated that for 28-day compressive strength, MRHA-10 and NRHA-8 mixes achieved the highest compressive strengths. However, NRHA-10 outperformed all other mixes in terms of strength at both 60 and 90 days. Praveenkumar and Vijayalakshmi (2019) evaluated the compressive strength of concrete mixes containing different combinations of NRHA and MRHA at 28 and 90 days. The NRHA-2.5 + MRHA-12.5 mix exhibited the best results, demonstrating a 38% increase in strength compared to the control mix. Ashok et al. (2017) investigated the use of NRHA. Their results showed a 17% improvement in strength for the mix containing 1.5% NRHA compared to the control mix. Notably, all NRHA blended mixes displayed improvements in strength at 7, 28, and 90 days compared to the control mix, (Fig. 8).

.

Praveenkumar and Vijayalakshmi (2019) observed that grinding RHA destroys the porous structure, leading to a reduced water requirement to achieve consistent workability in concrete mixes. However, it is important to note that compared to the control mix, both ground RHA and non-ground RHA generally require a higher water content or lead to a decrease in the workability. Furthermore, it has been reported that the grinding process can also increase both the initial and final setting times of the concrete due to the lower hydration rate associated with RHA. In response to the workability challenges, it is suggested to use superplasticizers to achieve the desired workability when incorporating RHA (Praveenkumar and Vijayalakshmi 2019).

The incorporation of NRHA synthesized from RHA significantly improved the resistance of the concrete to water penetration. This enhancement can be attributed to several mechanisms. Firstly, NRHA acts as a nucleation agent, promoting the formation of a denser and more uniform CSH gel with fewer pores (Balapour et al. 2017; Faried et al. 2021. Secondly, the nanoparticles effectively block the connections between capillary pores and channels within the concrete, hindering pathways for water absorption. Finally, the presence of NRHA reduces the interconnected porosity, thereby limiting the transport of ions through the concrete. The reduction in permeability corresponds with a decrease in the Coulombic resistance measured by the RCPT for mixes containing NRHA.

## Correlation in mechanical properties

The correlation of 28-day mechanical properties of RHA and nanomaterial-blended cement composites is presented herein. The following sections illustrate the variation of flexural and splitting tensile strength of composites compared to compressive strength.

### Flexural and compressive strength

**Fig. 9:**
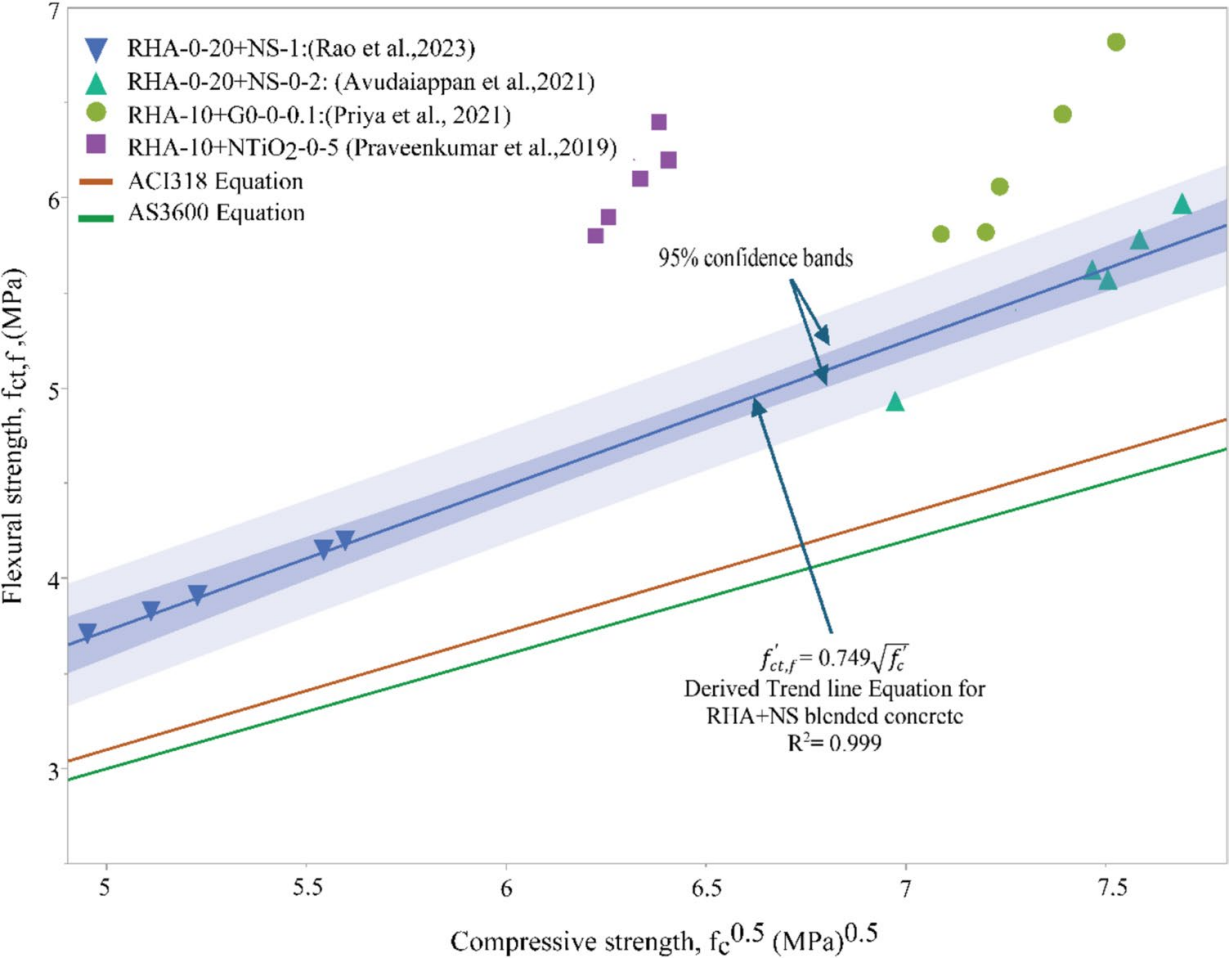
Relationship between compressive strength and flexural strength

Figure 9 illustrates the relationship between flexural and compressive strength from Rao et al. (2023): 0–20% RHA with 1% nanosilica, Avudaiappan et al. (2021): 0–20% RHA with 0–2% nanosilica, Priya et al. (2021): 10% RHA with 0–0.1% GO, Praveenkumar et al. (2019): 10% RHA with 0–5% TiO_2_ with the models provided in AS3600 (Eq. 1) and ACI 318 (Eq. 2).

Eq.1 $${f}_{ct,f}{\prime}= 0.60\sqrt{{f}_{c}{\prime}}$$

Eq.2 $${f}_{ct,f}{\prime}= 0.62\sqrt{{f}_{c}{\prime}}$$

The experimental data is significantly above the predicted relationship in both equations (Eqs.1 and 2). This illustrates flexural strength is considerably enhanced in blended (RHA and nanomaterials) concrete mixes. Furthermore, RHA with GO and N-TiO_2_ shows slightly greater improvement compared to RHA + NS-blended concrete. The linear regression line derived from the scatter plot of RHA + NS-blended concrete shown in Fig. 9 has a 0.99 coefficient of determination, indicating that a very high correlation between compressive and flexural strength remains for RHA modified concretes incorporating nanomaterials, similar to that observed in OPC concretes.

### Splitting tensile and compressive strength

**Fig. 10:**
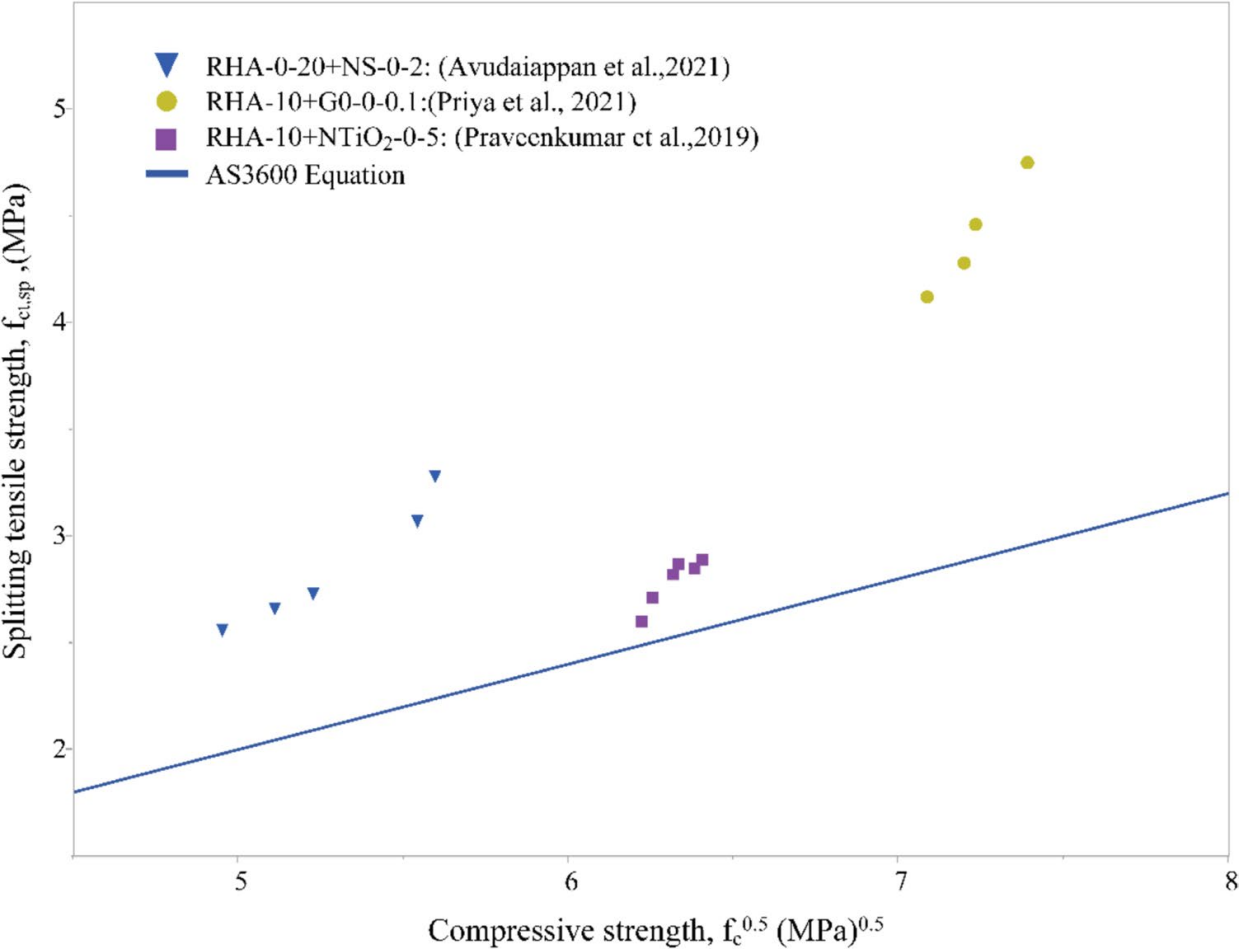
Relationship between compressive strength and splitting tensile strength

Figure 10 presents the scatter plot of splitting tensile strength versus compressive strength reported by Avudaiappan et al. (2021): 0–20% RHA with 0–2% nanosilica, Priya et al. (2021):10% RHA with 0–0.1% GO, and Praveenkumar et al. (2019): 10% RHA with 0–5% TiO_2_ with the derived equation (Eq. 3) in AS 3600 for the relationship between splitting tensile strength and compressive strength for PC concrete.

Eq.3 $${f}_{ct,f}{\prime}= 0.40\sqrt{{f}_{c}{\prime}}$$

Similar to flexural strength, splitting tensile strength is also greater in RHA nanomodified materials (Fig. 10). However, the relationship is more dependent on the nanomaterial added. The data illustrates greater improvement in RHA + NS- and RHA + GO-blended concretes, with RHA + NTiO_2_ only providing a slight improvement compared to OPC concretes.

## Discussion

While RHA-blended cement composites often demonstrate improved overall performance, their effectiveness is typically constrained to a relatively low level of replacement. This limitation stems primarily from reduced workability and the limited availability of the hydration products (CH) to react with their high silica content and form CSH. According to previous studies, replacement percentages exceeding 30% are not recommended, with 10–20% being optimal for both mechanical and durability performance. However, the review suggests that adopting ternary materials may enable higher replacement levels while reducing the overall environmental impact of the composite.

The physical and chemical properties of nanomaterials directly influence their characteristics and performance in composites (Abdalla et al. 2023; Srinivasan et al. 2024). In particular, the adoption of NS in composites, along with RHA as a ternary material, has received significant attention. The compressive strength of ternary cement composites exhibits an improvement of around 20–40%, with flexural and tensile strength following a similar trend. The combined effects of pozzolanic reaction and dense packing significantly enhance the performance of composites containing NS and RHA. Furthermore, the nanoparticles create nucleation sites for reaction and promote dense packing of the matrix, filling voids. These scenarios improve the early-age properties of both ternary and binary composites containing NS, making these composites ideal for structures requiring a rapid gain in mechanical properties (Chuah et al. 2014). However, the agglomeration of nanosilica particles due to strong Van der Waals forces reduces even distribution. Mixing processes such as sonication can be employed to address this issue.

Dense packing reduces the permeability of water, consequently lowering water absorption. Ternary composites exhibit lower water absorption compared to RHA-blended mixes due to the effective filling of voids by NS particles. Additionally, the presence of smaller diameter pores reduces their connectivity, further hindering water movement. Moreover, the even distribution of RHA contributes to a further decrease in water permeability. The chloride permeability of ternary blended composites also tends to decrease. Similarly, electric resistivity and resistance to chemical attack exhibit positive trends.

The combined effect of NS and RHA improves both the early-age and long-term mechanical and durability performance of composites. Compared to binary cement composites, ternary mixes containing NS exhibit a lower optimal replacement level. This phenomenon may be due to the combined effects of the pozzolanic reaction in RHA and the complex interactions occurring in ternary systems. However, standardized procedures for producing and incorporating NS-RHA blends in cement composites are required. The lack of such details hinders the widespread adoption of binary and ternary mixes.

GO has demonstrated greater enhancement in performance for binary and ternary cement composites compared to conventional concrete (Priya et al. 2021). The 2D shape of GO promotes a reinforcing effect that enhances the flexural and tensile strength of the composites. Investigation of the microstructure observes denser packing and fewer pores in composites containing both GNP and GO. Compared to other nanocarbon SCMs, GO exhibits less particle agglomeration due to good dispersibility in water or organic solvents. However, using a higher replacement level of GO increases agglomeration, necessitating dispersion methods like sonication to ensure even distribution.

The inclusion of NCaCO_3_ enhances the mechanical strength and durability of composites. While Divya et al. (2022) noted early-age performance in cement composites with NCaCO_3_, Rahbar et al. (2019) did not observe any effect. Both binary and ternary mixes containing NCaCO_3_ exhibit greater improvements in durability properties, including chloride resistance, electrical resistivity, and water absorption. However, further research is necessary to explore the optimal replacement level and the detailed characteristics of NCaCO_3_.

Mortar mixes containing 10–20% RHA and 3% NCuO also exhibit improved performance. This enhancement may be largely attributed to the filling effect of the nanoparticles. Electrical resistivity and chloride resistance exhibited similar trends. Studies on RHA + NTiO_2_-blended composite indicate improved workability with higher RHA and NTiO_2_ replacement levels. This is likely due to the nanoparticles filling the pores of RHA, as reported in Hassan et al (2013). This reduces water absorption. Similar improvements were observed in the mechanical properties. Notably, NTiO_2_ appears to act not only as a filling material but also as a pozzolanic material, further improving the composite matrix.

In addition, studies on nanosilica synthesized from RHA have shown that grinding alone leaves impurities in the nanosilica, hindering its effectiveness. Precipitation methods, on the other hand, can yield purer nanosilica. Smaller particle sizes and enhanced reactivity may enable lower replacement levels to achieve similar performance. Blending NRHA with RHA or other SCMs could promote sustainable cement composites by reducing the reliance on PC. Detailed studies are required to evaluate the effectiveness of these ternary mixes, including the sustainability of both the material processing techniques and the resulting composites.

**Fig. 11:**
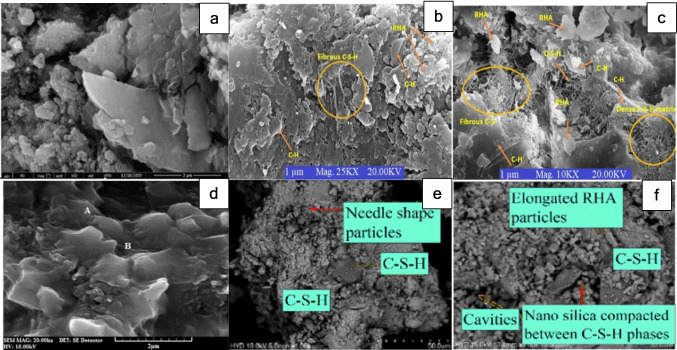
SEM micrographs of **a** NRHA3 (Faried et al. 2021), **b**,** c** RHA10, RHA10 + GO3 (Priya et al. 2021), **d** RHA10 + NcuO_3_ (Miyandehi et al. 2016), and **e**,** f** RHA10 + NS1 and RHA20 + NS1 (Rao et al. 2023)

It has been observed that the incorporation of nanomaterials tends to densify the microstructure, while some also engage in a pozzolanic reaction. RHA and GO blended mixes exhibit a denser microstructure compared to RHA-blended PC concrete. Furthermore, GO accelerates the hydration process, with GO blended composites tending to form fibrous CSH, which improves tensile and flexural properties, as shown in Fig. 11b, c. Composites with RHA and NS also exhibit a dense microstructure and higher CSH content due to the pozzolanic reaction of both the RHA and NS particles. Incorporating NCuO also results in the densification of the microstructure due to the filling effect and reduction in the number of pores and in pore size. This improves both mechanical and durability properties. Overall, this densification and reaction from the nanoparticles enhance the performance of the composites.

## Summary, conclusions, and recommendations

This review comprehensively examined concrete blended with RHA and nanomaterials. Table 1 summarizes the reported performance for each cement nanocomposite, detailing the replacement percentage and mixing methods, the properties of the blended composites, together with the associated issues and future recommendations.

**Table 1:** Summary of review

Replacement material	Replacement percentage	Mixing method	Fresh concrete properties	Mechanical properties	Durability properties	Microstructural properties	Issues	Future recommendations
RHA-blended	0–30%	Conventional mixing. Superplasticizers are used to improve workability	Reduced workability	Up to 32% improvement in compressive strength	Reduced chloride permeability. No significant impact on water absorption	Increased CSH formation due to the pozzolanic reaction	Variability of material properties due to the production process impacts composite performance	Development of databases and establish relationships for industry adoption
RHA + nanosilica	0–3% nanosilica	Sonication is used to mix the nanosilica	Reduced workability due to smaller particle size and higher specific surface area	Up to 29% improvement in compressive strength	Reduced water absorption and chloride permeability; improved resistance to acid attack	A denser microstructure with increased CSH Ternary mixes show higher CSH content	Agglomeration of nanosilica	-
RHA + nanocarbon	0–0.1% nanocarbon	Sonication process. Polycarboxylic-based superplasticizers are used	Reduced workability	Improved compressive, flexural, and splitting tensile strength	Reduced chloride permeability	A denser microstructure CSH may react with carboxylic groups in the nanocarbon	Agglomeration of nanomaterials	Requires further studies to identify short- and long-term performance
RHA + nano-CaCO_3_	0–1.5% nano-CaCO_3_	Conventional mixing with superplasticizers	Reduced workability	Up to 15% increase in compressive strength	Reduced water absorption; increased electrical resistivity and chloride resistance	-	-	Requires further studies to evaluate mechanical, durability, and microstructural performance
RHA + nano-CuO	0–3% nano-CuO	Conventional mixing	Does not vary significantly with the NCuO content	Up to 15% improvement in compressive strength	Enhanced chloride resistance and electrical resistivity	-	-	Requires further studies to evaluate mechanical, durability, and microstructural performance
RHA + nano-TiO_2_	1–5% nano-TiO_2_	-	Workability increases with NTiO_2_ content	Improved compressive, flexural, and splitting tensile strength	Reduced water absorption, increased resistance to acid attack, and reduced chloride permeability	Densifies the microstructure and contributes to the pozzolanic reaction	-	Requires further studies to evaluate overall performance
Nano-RHA	0–10%	Conventional mixing with superplasticizers	Workability is improved compared to RHA	Up to 38% increase in compressive strength	Reduced water absorption	Dense microstructure with reduced interconnected porosity	-	Require further studies to evaluate short and long-term mechanical and durability performance

The following conclusions are drawn from the review:

- Due to reactive silica in RHA, substitution of PC by up to 30% can enhance or, as a minimum, maintain performance of cement composites. The optimal replacement level is between 10 and 20%.
- Among the mixing methods for cement nanocomposites, sonication and the use of polycarboxylic-based superplasticizers ensure good mixing and prevent the agglomeration of nanomaterials. However, the practicality of applying these techniques should be evaluated. Extensive research on RHA and nanosilica-blended composites has demonstrated improvements in mechanical and durability properties. The higher reactivity of nanoparticles contributes to early-age enhancement. However, nanoparticle agglomeration limits the extent of this improvement.
- Studies on RHA and nanocarbon-blended composites are limited. However, GO, in particular, exhibits lower agglomeration effects due to its water solubility. Furthermore, significant enhancements in flexural and splitting tensile strengths were observed, potentially attributed to the 2D morphology of GO particles providing a reinforcing effect.
- NCaCO_3_ demonstrates significant enhancement contributing to hydration kinetics and filling effects, while NTiO_2_ and NCuO enhance performance primarily through the filling effect of the nanoparticles.
- Further research is necessary to elucidate the reaction mechanisms and establish the relationships between performance and SCM replacement levels to develop optimal blended mixes and facilitate their adoption in the construction sector. The environmental impact and whole-life cost of these composites should be explored to determine their feasibility.

## Data Availability

All data generated or analyzed during this study are included in this published article and will be made available upon reasonable request.
